# Hemoglobin glycation index and prognosis after successful chronic total occlusion percutaneous coronary intervention: a retrospective cohort study

**DOI:** 10.3389/fendo.2026.1945216

**Published:** 2026-09-10

**Authors:** Song Wen, Zehan Huang, Lingbing Meng, Qiheng Wan, Xingjie Huang, Yuqing Huang, Bin Zhang, Jing Wang

**Affiliations:** 1 Department of Cardiology, Guangdong Cardiovascular Institute, Guangdong Provincial People’s Hospital, Guangdong Academy of Medical Sciences, Southern Medical University, Guangzhou, Guangdong, China; 2 Department of Cardiology, Fuwai Hospital, National Center for Cardiovascular Diseases, Chinese Academy of Medical Sciences and Peking Union Medical College, Beijing, China; 3 Department of Cardiology, The Second Affiliated Hospital of Guilin Medical University, Guilin, Guangxi, China; 4 Department of Cardiology, Shenzhen Luohu Hospital Group Luohu People’s Hospital, The Third Affiliated Hospital of Shenzhen University, Shenzhen, China

**Keywords:** chronic total occlusion, glycemic variability, hemoglobin glycation index, percutaneous coronary intervention, prognosis

## Abstract

**Background:**

Even after technically successful percutaneous coronary intervention (PCI) for chronic total occlusion (CTO), patients face persistent excess cardiovascular risk. The hemoglobin glycation index (HGI), a marker of interindividual glycation heterogeneity, has not been studied in this setting.

**Methods:**

We enrolled 1, 513 patients who had undergone successful CTO-PCI between 2011 and 2023 at Guangdong Provincial People’s Hospital. HGI was computed as the difference between the measured HbA1c and the value predicted from fasting plasma glucose using a cohort-derived linear equation. The primary composite outcome included cardiovascular death, non-fatal myocardial infarction, and stroke (cardiovascular events, CVEs). Secondary outcomes were all-cause and cardiovascular death. Multivariable Cox regression and restricted cubic splines were applied to assess associations.

**Results:**

During a median 810-day follow-up, 83 (5.5%) all-cause deaths, 53 (3.5%) cardiovascular deaths, and 73 (4.8%) CVEs occurred. After full adjustment, Each 1-standard deviation increment in HGI conferred an independent hazard of 1.34 (95% CI 1.14–1.58) for all-cause mortality, 1.52 (95% CI 1.25–1.83) for cardiovascular mortality, and 1.42 (95% CI 1.15–1.61) for CVEs. Compared with the lowest tertile, patients in the highest HGI tertile exhibited a 2.41-fold (95% CI 1.37–4.25, P = 0.002), 4.13-fold (95% CI 1.91–8.94, P<0.001), and 2.73-fold (95% CI 1.47–5.07, P = 0.001) higher risk of all-cause mortality, cardiovascular mortality, and CVEs, respectively. RCS analysis revealed a linear dose-response relationship, with a secondary exploratory threshold effect identified at HGI = –0.15 for CVEs (HR 2.05, 95% CI 1.22–3.44).

**Conclusions:**

In this retrospective cohort study, elevated HGI was independently and linearly associated with heightened long-term adverse clinical outcomes after successful CTO-PCI. These findings suggest that HGI may have potential as a prognostic marker for risk stratification in this population; however, prospective validation is required before clinical application.

## Introduction

Chronic total occlusion (CTO) constitutes a frequent and technically demanding subset of coronary artery disease (CAD), detected in 15% to 30% of diagnostic angiograms (1, 2). Successful revascularization by PCI affords symptomatic relief, reduces the need for subsequent coronary artery bypass grafting (CABG), and improves long-term survival (3, 4). Nevertheless, even after an angiographically and procedurally successful CTO-PCI, patients encounter appreciable residual risk of recurrent events and death (5). Hence, there is a pressing need for accessible biomarkers that refine post-procedural risk appraisal and inform tailored secondary prevention.

Glycemic control remains a cornerstone of secondary cardiovascular prevention across diabetic and non-diabetic populations. HbA1c is the established benchmark for chronic glycemia and has been consistently correlated with cardiovascular outcomes in broad cohorts and in patients with established CAD (6, 7). However, HbA1c represents an integrated average and does not account for substantial person-to-person variation in hemoglobin glycation at equivalent fasting glucose levels (8). The hemoglobin glycation index (HGI)—defined as the discrepancy between measured HbA1c and the level predicted from FPG—has been proposed as a quantitative indicator of this individual glycation propensity (9). Accumulating evidence suggests that HGI predicts microvascular and macrovascular complications of diabetes, with higher values signifying enhanced tissue glycation and oxidative burden (10, 11).

Although the cardiovascular relevance of HGI is increasingly recognized, its prognostic utility in the specific context of successful CTO-PCI has not been investigated. Earlier reports have primarily addressed general diabetic cohorts, patients with acute coronary syndromes, or those undergoing non-CTO-PCI (12–14), leaving a knowledge gap for this high-risk revascularization subgroup. Moreover, whether HGI contributes incremental risk discrimination beyond traditional glycemic measures and established cardiovascular risk factors after CTO recanalization remains uncertain. Given the distinctive pathophysiology of CTO, marked by chronic ischemia, collateral network remodeling, and a heightened pro-inflammatory and pro-thrombotic milieu (15, 16), the prognostic meaning of HGI in this setting could differ from that observed in other CAD populations.

In this retrospective cohort study, we tested the hypothesis that higher HGI is independently linked to worse long-term outcomes after successful CTO-PCI. Our findings may help refine post-procedural risk assessment and guide personalized secondary prevention.

## Methods

### Study population and design

This retrospective, single-center analysis initially identified 1, 756 consecutive adults who underwent PCI for at least one angiographically documented CTO at Guangdong Provincial People’s Hospital between February 2011 and December 2023. After removing 72 cases of procedural failure, 79 with incomplete HGI or essential covariate data, and 100 lost to follow-up, the final analytical sample comprised 1, 513 patients.

This study represents a pre-specified secondary analysis of the Guangdong Provincial People’s Hospital CTO-PCI registry, a continuous clinical quality improvement database initiated in January 2011. The registry protocol, including the present secondary analysis, was approved by the Guangdong Provincial People’s Hospital Institutional Review Board (approval number: GDREC2017196H[R1]). This approval explicitly authorized the retrospective analysis of all consecutive patients treated at our institution from January 2011 onward. All data elements were collected prospectively as part of routine clinical care according to standardized departmental protocols, and the Institutional Review Board confirmed that the data collection and follow-up procedures for the entire study period complied with the ethical standards of the Declaration of Helsinki. Given the retrospective design, the use of pre-existing routinely collected clinical data, and the minimal risk to patients, the ethics committee granted a waiver of informed consent. No additional ethical approval or protocol amendment was required for this secondary analysis.

### Data collection and definitions

Indications for CTO-PCI included refractory angina or dyspnea despite optimized medical therapy, or documented inducible ischemia with preserved myocardial viability in the CTO territory (17, 18). The presence and extent of inducible ischemia were assessed using non-invasive stress imaging, including single-photon emission computed tomography (SPECT), cardiac magnetic resonance (CMR) with adenosine stress perfusion, or quantitative positron emission tomography (PET) perfusion. Myocardial viability was evaluated using CMR with late gadolinium enhancement (LGE), where segments with ≤50% transmural LGE were considered viable, and <25% was regarded as predictive of functional recovery. Additional viability assessments included dobutamine stress echocardiography (contractile reserve) and 18F-FDG PET (perfusion-metabolism mismatch). In selected cases with subtotal lesions, invasive FFR was used to corroborate hemodynamic significance. All interventions were performed by high-volume operators (each with a lifetime experience of >300 CTO-PCI procedures and an annual caseload of ≥50 as primary operator). A CTO was defined as complete antegrade luminal obstruction with an estimated occlusion duration of at least 3 months, based on clinical history, prior MI records, and previous angiographic studies (19). Technical success mandated a final TIMI flow grade ≥2 in all distal vessels ≥2.5 mm, accompanied by residual stenosis <30%. Procedural success required technical success without the occurrence of in-hospital major adverse events (death, MI, or target-vessel revascularization) (19).

**Figure 1:**
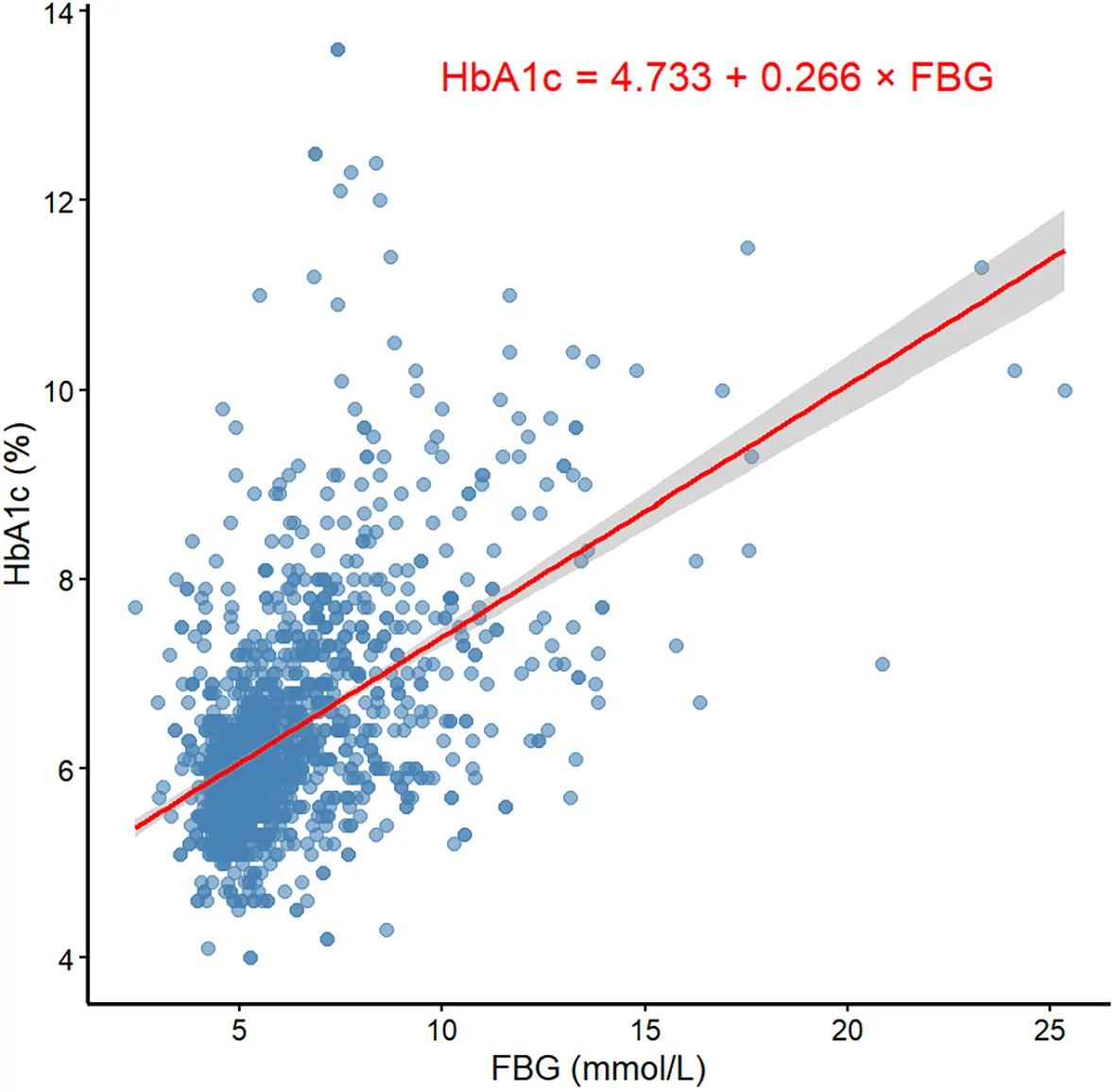
Regression of fasting plasma glucose on glycated haemoglobin. The oblique line represents the simple linear regression line of the equation: HbA1c (%) = 0.266 × FPG (mmol/L) + 4.733.

We retrospectively compiled baseline demographic and clinical data, including age, sex, comorbidities, smoking habits, and prior MI or revascularization events. Clinical information comprised physical examination findings, routine laboratory tests, imaging assessments, and discharge medications. Fasting venous blood samples were drawn on the morning of the day prior to or the day of the CTO PCI procedure, after ≥12 hours of overnight fasting, and analyzed at the institution’s central laboratory under strict quality-assurance protocols. The measured parameters covered complete blood count, lipid panel, FPG, haemoglobin A1c (HbA1c), alanine aminotransferase (ALT), aspartate aminotransferase (AST), albumin (ALB), creatinine, uric acid, troponin T (TnT), N-terminal pro-B-type natriuretic peptide (NT-proBNP), creatine kinase (CK), CK-MB, and C-reactive protein (CRP). LVEF was obtained via echocardiography during the same pre-procedural hospitalization using the biplane Simpson method (20). DM was diagnosed based on prior documentation, use of glucose-lowering agents, or laboratory thresholds (FPG ≥7.0 mmol/L, HbA1c ≥6.5%, or 2-hour plasma glucose ≥11.1 mmol/L). Hypertension was defined by a documented history, current anti-hypertensive treatment, or a blood pressure reading ≥140/90 mmHg (18). To derive HGI, we first constructed a linear regression equation from the entire cohort’s FPG and HbA1c data: Predicted HbA1c (%) = 0.266 × FPG (mmol/L) + 4.733. HGI was then computed for each individual as the measured HbA1c minus the predicted value (**Figure 1**) (11–14).

### Follow-up and clinical endpoints

The median observation period was 810 (interquartile range, 409–1230)days. The primary outcome was a composite of cardiovascular death, non-fatal MI, and non-fatal stroke, termed cardiovascular events (CVEs). Secondary outcomes comprised all-cause mortality and cardiovascular death separately. Following discharge, all patients were scheduled for routine outpatient visits at 1, 3, and 6 months, and annually thereafter. For patients unable to attend scheduled visits, structured telephone interviews were conducted by trained research personnel using a standardized questionnaire. Clinical outcomes were further ascertained through electronic health record review, which captures all hospitalizations and emergency visits within our healthcare network. The follow-up intervals and assessment protocols were standardized across all participants, and all suspected events were independently adjudicated by two experienced cardiologists (20).

### Statistical analysis

Continuous variables are reported as medians with interquartile ranges, and categorical variables as frequencies and percentages. Between-group comparisons across HGI tertiles were performed using the Kruskal-Wallis test for continuous measures and the chi-square or Fisher’s exact test for categorical variables.

Cumulative event rates by HGI tertile were depicted using Kaplan-Meier curves, with differences assessed by the log-rank test. Multivariable Cox proportional-hazards models were employed to estimate hazard ratios and 95% confidence intervals for HGI, analysed both as a continuous variable (per 1-SD increment) and as tertiles (with the lowest tertile as reference). Covariates were selected *a priori* based on established cardiovascular risk factors, prior CTO-PCI prognostic studies, and biological plausibility, to minimize the risk of spurious associations and overfitting. Three sequential adjustment schemes were applied: Model 1 was unadjusted; Model 2 accounted for age, sex, smoking, hypertension, prior MI, and prior stroke; Model 3 additionally included multi-vessel disease, LM/LAD CTO, LDL-C, TG, NT-proBNP, troponin T, creatinine, uric acid, CRP, LVEF, β-blocker, CCB, and ACEI/ARB. The proportional-hazards assumption was checked via Schoenfeld residuals and was satisfied for all models. Dose-response relationships were explored using restricted cubic splines (RCS) with knots placed at the 30th, 60th, and 90th percentiles. To explore potential threshold effects, we performed a piecewise Cox regression analysis with a grid search across the observed HGI range (5th to 95th percentile) to identify the cut-point that maximized the model log-likelihood. The optimal cut-point was then evaluated in a multivariable Cox model with HGI as a binary variable, adjusted for the same covariates as Model 3.

Subgroup analyses with formal interaction testing were performed for age, smoking, hypertension, diabetes, prior MI, LDL-C, and LVEF. Sensitivity analyses were performed to assess the robustness of the primary findings. These included: (1) exclusion of patients with a LVEF <30%; (2) exclusion of patients with a follow-up duration <90 days; (3) exclusion of patients with prior CABG; (4) additional adjustment for diabetes; and (5) to further address the concern regarding potential overfitting given the limited number of outcome events, we constructed a parsimonious multivariable Cox model that included only covariates with P < 0.05 in univariable analysis, while forcing HGI to remain in the model as the primary exposure variable. Using cardiovascular events as the endpoint, 10 covariates met the screening threshold: age, hypertension, diabetes, prior stroke, NT-proBNP, UA, TNT, creatinine, LVEF, and CRP.

A two-sided P value <0.05 was considered to indicate statistical significance. All statistical analyses were carried out using R software, version 4.1.2 (R Foundation for Statistical Computing).

## Results

### Baseline characteristics

The study population (N = 1, 513) had a median age of 59.0 (52.0–67.0) years, with 1, 386 (91.6%) male participants. **Table 1** presents baseline features according to HGI tertile. Patients in higher tertiles tended to be older, had higher systolic blood pressure, and showed a greater prevalence of both DM and hypertension (all P<0.05). Many laboratory indices, including haemoglobin, platelet count, white blood cell count, HDL-C, LDL-C, TG, TC, FPG, HbA1c, creatinine, blood urea nitrogen, albumin, NT-proBNP, TNT, CRP, and LVEF, varied significantly across tertiles (all P<0.05). In contrast, the use of statins, β-blockers, dual antiplatelet therapy, CCB, or ACEI/ARB was similar among the three groups (all P>0.05).

**Table 1:** Baseline characteristics grouped by HGI levels.

Variables	All (N = 1513)	T1≤-0.55 (N = 505)	-0.55<T2<0.36 (N = 504)	T3≥0.36 (N = 504)	P value
Age, years	59.00 (52.00, 67.00)	58.00 (51.00, 65.00)	61.00 (52.00, 67.00)	60.50 (53.00, 68.00)	<0.001
Male, n (%)	1, 386.0 (91.6%)	474.0 (93.9%)	458.0 (90.9%)	454.0 (90.1%)	0.074
SBP, mmHg	130.00 (119.00, 143.00)	127.00 (117.00, 138.00)	131.00 (121.50, 144.00)	132.00 (117.50, 146.00)	0.001
DBP, mmHg	78.00 (70.00, 85.00)	78.00 (69.00, 85.00)	78.00 (71.00, 85.50)	78.00 (70.00, 85.00)	0.5
Heart rate, beats/min	74.00 (67.00, 82.00)	74.00 (67.00, 81.00)	73.00 (66.00, 82.00)	75.00 (68.00, 83.00)	0.059
Current smoker, n (%)	588.0 (38.9%)	196.0 (38.8%)	198.0 (39.3%)	194.0 (38.5%)	>0.9
Hypertension, n (%)	902.0 (59.6%)	277.0 (54.9%)	305.0 (60.5%)	320.0 (63.5%)	0.018
Diabetes, n (%)	602.0 (39.8%)	111.0 (22.0%)	125.0 (24.8%)	366.0 (72.6%)	<0.001
Dyslipidemia, n (%)	423.0 (28.0%)	139.0 (27.5%)	146.0 (29.0%)	138.0 (27.4%)	0.8
Prior MI, n (%)	237.0 (15.7%)	79.0 (15.6%)	77.0 (15.3%)	81.0 (16.1%)	>0.9
Prior PCI, n (%)	757.0 (50.0%)	249.0 (49.3%)	258.0 (51.2%)	250.0 (49.6%)	0.8
Prior CABG, n (%)	36.0 (2.4%)	11.0 (2.2%)	7.0 (1.4%)	18.0 (3.6%)	0.071
Prior stroke, n (%)	75.0 (5.0%)	16.0 (3.2%)	28.0 (5.6%)	31.0 (6.2%)	0.069
Laboratory results					
Hb, g/L	135.00 (125.00, 146.00)	137.00 (125.00, 147.00)	137.00 (127.00, 146.00)	132.00 (122.00, 144.00)	0.001
WBC, 10^9^/L	6.96 (5.84, 8.25)	6.80 (5.78, 8.07)	6.79 (5.76, 8.13)	7.20 (5.99, 8.54)	0.002
PLT, 10^9^/L	217.00 (180.00, 263.00)	208.00 (171.00, 254.00)	220.00 (190.00, 258.00)	224.00 (180.00, 271.50)	0.001
LDL, mmol/L	2.39 (1.87, 2.95)	2.29 (1.81, 2.87)	2.43 (1.93, 2.94)	2.44 (1.91, 3.01)	0.046
HDL, mmol/L	0.93 (0.82, 1.07)	0.93 (0.83, 1.06)	0.96 (0.85, 1.10)	0.91 (0.79, 1.04)	<0.001
TC, mmol/L	3.87 (3.20, 4.60)	3.77 (3.10, 4.48)	3.91 (3.25, 4.65)	3.91 (3.21, 4.69)	0.032
TG, mmol/L	1.47 (1.06, 2.09)	1.46 (1.05, 2.05)	1.43 (1.03, 1.95)	1.58 (1.12, 2.28)	0.008
FBG, mmol/L	5.50 (4.85, 6.90)	5.50 (4.92, 7.20)	5.22 (4.77, 6.13)	5.90 (4.91, 7.40)	<0.001
HbA1c, %	6.10 (5.70, 6.80)	5.50 (5.30, 5.80)	6.00 (5.80, 6.27)	7.20 (6.50, 7.90)	<0.001
Creatinine, µmol/L	84.00 (72.90, 100.19)	85.00 (73.33, 98.02)	80.97 (71.63, 97.67)	86.29 (73.00, 107.26)	0.025
BUN, µmol/L	5.59 (4.52, 6.83)	5.46 (4.46, 6.56)	5.53 (4.53, 6.66)	5.85 (4.70, 7.39)	0.001
UA, μmol/L	404.90 (337.00, 478.00)	405.00 (342.00, 478.00)	403.10 (333.70, 483.70)	406.10 (334.30, 472.44)	>0.9
ALT, U/L	22.00 (16.00, 32.00)	23.00 (15.00, 31.00)	21.00 (16.00, 31.00)	22.00 (15.00, 32.50)	0.9
AST, U/L	22.00 (18.00, 27.00)	22.00 (18.00, 27.00)	21.50 (18.00, 27.00)	21.00 (17.00, 27.00)	0.050
ALB, g/L	38.80 (36.56, 41.00)	39.10 (36.76, 41.25)	38.93 (36.82, 40.91)	38.36 (35.97, 40.45)	0.002
NT-proBNP, pg/ml	229.40 (73.40, 749.70)	229.30 (73.40, 721.00)	167.70 (63.35, 610.80)	327.90 (92.25, 1, 086.63)	<0.001
TNT, pg/ml	17.20 (9.70, 41.24)	16.20 (9.00, 47.20)	16.20 (9.10, 32.25)	19.55 (11.95, 45.55)	<0.001
CK, U/L	87.00 (63.00, 122.00)	89.00 (66.00, 124.00)	86.50 (65.00, 118.50)	82.00 (59.00, 120.50)	0.070
CK-MB, U/L	10.20 (10.00, 13.00)	10.10 (10.00, 12.70)	10.00 (10.00, 13.00)	10.80 (10.00, 13.20)	0.094
CRP, mg/L	1.97 (0.50, 6.20)	1.86 (0.50, 6.10)	1.79 (0.50, 5.40)	2.42 (0.50, 7.25)	0.014
LVEF, %	58.00 (46.00, 63.00)	59.00 (46.00, 63.00)	59.00 (50.00, 63.50)	54.00 (43.00, 63.00)	0.003
HGI	-0.15 (-0.55, 0.36)	-0.75 (-1.07, -0.55)	-0.15 (-0.27, 0.00)	0.67 (0.36, 1.26)	<0.001
Angiographic characteristics					
LM/LAD CTO, n (%)	716 (47.3%)	239 (47.9%)	249 (49.4%)	225 (44.6%)	0.4
Multi-vessel disease, n (%)	1, 354.0 (89.5%)	453.0 (89.7%)	440.0 (87.3%)	461.0 (91.5%)	0.10
Medications					
DAPT, n (%)	1, 477.0 (97.6%)	492.0 (97.4%)	493.0 (97.8%)	492.0 (97.6%)	>0.9
Statin, n (%)	1, 494.0 (98.7%)	496.0 (98.2%)	501.0 (99.4%)	497.0 (98.6%)	0.2
β‐Blocker, n (%)	1, 211.0 (80.0%)	411.0 (81.4%)	394.0 (78.2%)	406.0 (80.6%)	0.4
CCB, n (%)	318.0 (21.0%)	91.0 (18.0%)	110.0 (21.8%)	117.0 (23.2%)	0.11
ACEI/ARB, n (%)	902.0 (59.7%)	296.0 (58.7%)	292.0 (57.9%)	314.0 (62.3%)	0.3
Clinical outcomes					
All-cause mortality, n (%)	83 (5.5%)	20.0 (4.0%)	18.0 (3.6%)	45.0 (8.9%)	<0.001
Cardiovascular mortality, n (%)	53 (3.5%)	10.0 (2.0%)	11.0 (2.2%)	32.0 (6.3%)	<0.001
Cardiovascular events, n (%)	73 (4.8%)	17.0 (3.4%)	16.0 (3.2%)	40.0 (7.9%)	17.0 (3.4%)
Non−fatal MI, n (%)	9.0 (0.6%)	5.0 (1.0%)	3.0 (0.6%)	1.0 (0.2%)	0.3
Non−fatal Stroke, n (%)	14.0 (0.9%)	2.0 (0.4%)	5.0 (1.0%)	7.0 (1.4%)	0.2

HGI, hemoglobin glycation index; LVEF, left ventricular ejection fraction; PCI, percutaneous coronary intervention; CABG, coronary artery bypass grafting; SBP, systolic blood pressure; DBP, diastolic blood pressure; MI, myocardial infarction; TC, total cholesterol; HDL-C, high-density lipoprotein cholesterol; LDL-C, low-density lipoprotein cholesterol; TG, triglycerides; FBG, fasting blood glucose; CRP, C-reactive protein; BUN, blood urea nitrogen; UA, uric acid; HbA1c, hemoglobin A1c; Hb, hemoglobin; WBC, white blood cell; PLT, platelet; ALT, Alanine Aminotransferase; BNP, brain natriuretic peptide; TNT, Troponin T; CK, creatine kinase; CK-MB, creatine kinase-MB; AST, aspartate aminotransferase; ALB, albumin;LAD, left Anterior descending artery; LM, left main coronary artery; DAPT, dual antiplatelet therapy; CCB, calcium channel blocker; ACEI, angiotensin-converting enzyme inhibitor; ARB, angiotensin II receptor blocker.

### Association of HGI with clinical outcomes in patients with successful CTO PCI

**Figure 2:**
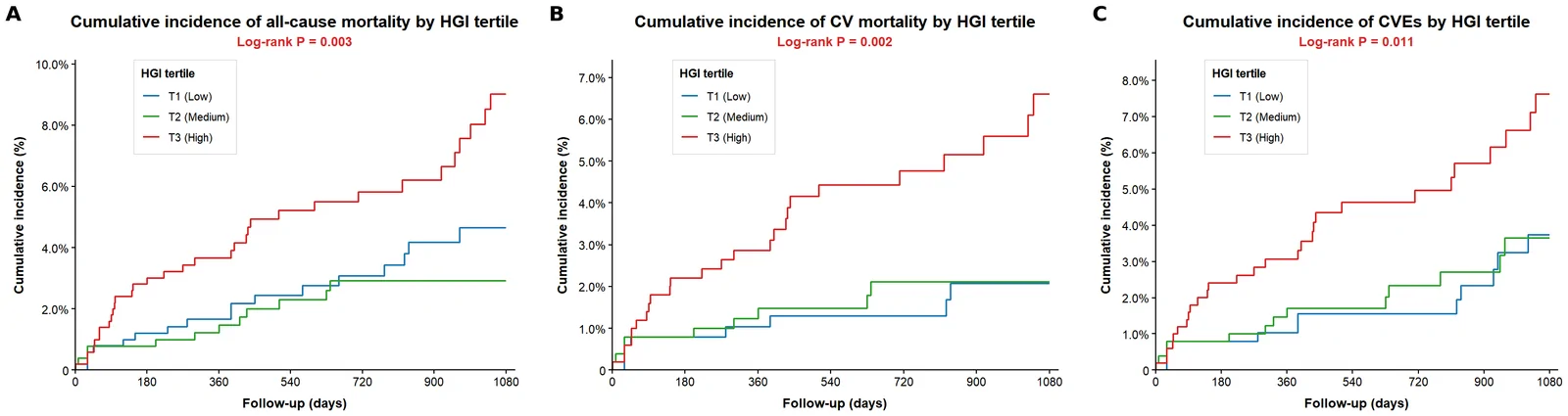
Kaplan-Meier curves of the cumulative incidence of **(A)** all−cause mortality, **(B)** cardiovascular mortality and **(C)** cardiovascular events according to HGI.

Over a median follow-up of 810 days, we recorded 83 all-cause deaths (5.5%), 53 cardiovascular deaths (3.5%), and 73 CVEs (4.8%). Kaplan-Meier plots demonstrated clear and significant divergence in the cumulative incidence of all three endpoints according to HGI tertile (log-rank P<0.05; **Figure 2**). In unadjusted analyses, each 1-standard deviation (SD) increment in HGI was associated with a 21% higher risk of all-cause mortality (HR 1.21, 95% CI 1.02-1.44, P = 0.028), 35% higher risk of cardiovascular mortality (HR 1.35, 95% CI 1.12-1.62, P = 0.001), and 28% higher risk of CVEs (HR 1.28, 95% CI 1.07-1.52, P = 0.006). After full adjustment (Model 3), the corresponding HRs were 1.34 (95% CI 1.14-1.58, P<0.001), 1.52 (95% CI 1.25-1.83, P<0.001), and 1.42 (95% CI 1.15-1.61, P<0.001), respectively.

**Figure 3:**
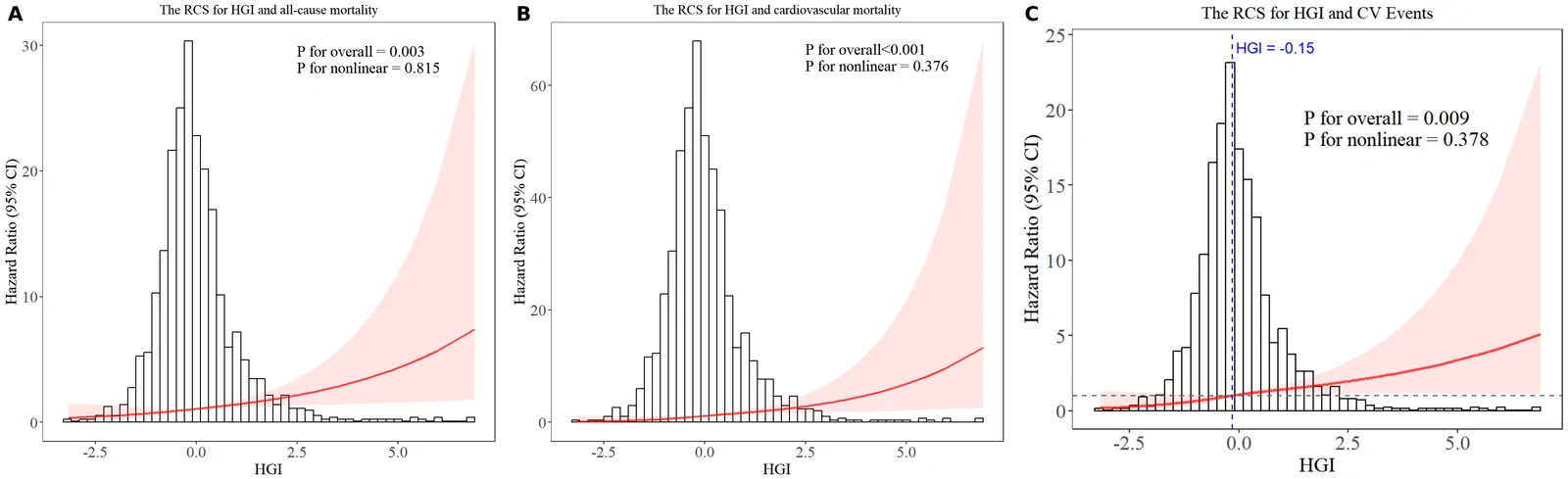
RCS analysis of the HGI for incidence of **(A)** all−cause mortality, **(B)** cardiovascular mortality, and **(C)** cardiovascular events.

When HGI was assessed by tertiles, relative to the lowest tertile (T1), the highest tertile (T3) conferred a 2.41-fold greater all-cause mortality (HR 2.41, 95% CI 1.37-4.25, P = 0.002), a 4.13-fold higher cardiovascular mortality (HR 4.13, 95% CI 1.91-8.94, P<0.001), and a 2.73-fold elevated risk of CVEs (HR 2.73, 95% CI 1.47-5.07, P = 0.001) (**Table 2**). RCS analysis revealed a linear association between HGI and all-cause mortality, cardiovascular mortality, and major cardiovascular events (all P_for non-linearity_ >0.05) (**Figure 3**). In a secondary exploratory analysis, we performed threshold effect analysis using piecewise Cox regression with grid search, which identified an cut-point at HGI = –0.15 for CVEs. Patients with HGI > –0.15 had a 2.05-fold higher risk of CVEs (HR 2.05, 95% CI 1.22–3.44, P = 0.006) compared with those with HGI ≤ –0.15 after comprehensive multivariable adjustment (**Table 3**).

**Table 2:** Associations between HGI and clinical outcomes in participants after successful CTO PCI.

Outcomes	Model 1		Model 2		Model 3	
	HR (95% CI)	*P*-value	HR (95% CI)	*P*-value	HR (95% CI)	*P*-value
All-cause mortality						
Per 1-SD increase in HGI	1.21 (1.02-1.44)	0.028	1.22 (1.02-1.46)	0.032	1.34 (1.14-1.58)	<0.001
T1	Reference		Reference		Reference	
T2	0.89 (0.47-1.69)	0.732	0.78 (0.41-1.47)	0.435	1.07 (0.54-2.12)	0.841
T3	2.11 (1.25-3.58)	0.005	1.83 (1.08-3.10)	0.026	2.41 (1.37-4.25)	0.002
Cardiovascular mortality						
Per 1-SD increase in HGI	1.35 (1.12-1.62)	0.001	1.38 (1.13-1.67)	0.001	1.52 (1.25-1.83)	<0.001
T1	Reference		Reference		Reference	
T2	1.09 (0.46-2.58)	0.836	0.98 (0.41-2.31)	0.960	1.42 (0.57-3.54)	0.453
T3	3.05 (1.50-6.20)	0.002	2.63 (1.29-5.36)	0.008	4.13 (1.91-8.94)	<0.001
Cardiovascular events						
Per 1-SD increase in HGI	1.28 (1.07-1.52)	0.006	1.28 (1.07-1.54)	0.008	1.36 (1.15-1.61)	<0.001
T1	Reference		Reference		Reference	
T2	1.01 (0.51-2.02)	0.974	0.89 (0.45-1.79)	0.754	1.25 (0.60-2.60)	0.546
T3	2.36 (1.32-4.21)	0.004	2.01 (1.12-3.60)	0.019	2.73 (1.47-5.07)	0.001

Model 1 adjust for: None.

Model 2 adjust for: age, sex, smoking, hypertension, prior MI, and prior stroke.

Model 3 adjust for: age, sex, smoking, hypertension, prior MI, prior stroke, multi−vessel disease, LM/LAD CTO, LDL−C, TG, NT-proBNP, TNT, creatinine, UA, CRP, LVEF, β‐Blocker, CCB, and ACEI/ARB.

HR, hazard ratio; CI, confidence interval; HGI, hemoglobin glycation index; CTO, chronic total occlusion; PCI, percutaneous coronary intervention.

**Table 3:** Associations between HGI and CVEs in participants after successful CTO PCI based on the cut-point of HGI at -0.15.

Cardiovascular events	Model 1		Model 2		Model 3	
	HR (95% CI)	*P*-value	HR (95% CI)	*P*-value	HR (95% CI)	*P*-value
HGI≤-0.15	Reference		Reference		Reference	
HGI>-0.15	1.73 (1.06-2.81)	0.028	1.57 (0.97-2.56)	0.068	2.05 (1.22-3.44)	0.006

Model 1 adjust for: None.

Model 2 adjust for: age, sex, smoking, hypertension, prior MI, and prior stroke. .

Model 3 adjust for: age, sex, smoking, hypertension, prior MI, prior stroke, multi−vessel disease, LM/LAD CTO, LDL−C, TG, NT-proBNP, TNT, creatinine, UA, CRP, LVEF, β‐Blocker, CCB, and ACEI/ARB.

HR, hazard ratio; CI, confidence interval; HGI, hemoglobin glycation index; CTO, chronic total occlusion; PCI, percutaneous coronary intervention.

### Subgroup analyses

The positive associations were generally consistent across all predefined subgroups (**Table 4**; **Supplementary Tables S1**, **S2**). Notably, a significant interaction between hypertension and HGI emerged for cardiovascular mortality (P interaction=0.046) and CVEs (P interaction=0.013), with stronger effects observed in non-hypertensive individuals; however, no such interaction was detected for all-cause mortality (P interaction=0.113).

**Table 4:** Subgroup analysis of association of HGI and CVEs in participants with successful CTO PCI.

Subgroups		CVEs	N	HR (95% CI)	P value	P for interaction
Age	≤60	25	814	1.44 (1.04-2.01)	0.030	0.426
	>60	48	699	1.42 (1.12-1.80)	0.004	0.426
Diabetes	Yes	38	602	1.29 (1.04-1.59)	0.018	0.564
	No	35	911	1.63 (0.86-3.08)	0.137	0.564
Hypertension	Yes	56	902	1.25 (1.01-1.55)	0.045	0.013
	No	17	611	2.19 (1.46-3.29)	<0.001	0.013
Prior MI	Yes	20	237	1.73 (1.07-2.79)	0.024	0.479
	No	53	1, 276	1.32 (1.09-1.61)	0.005	0.479
Current Smoking	Yes	25	588	1.27 (0.86-1.90)	0.233	0.838
	No	48	925	1.41 (1.15-1.72)	<0.001	0.838
LDL-C	≥2.6	30	590	1.27 (0.99-1.61)	0.058	0.750
	<2.6	43	923	1.48 (1.08-2.02)	0.014	0.750
LVEF	<60	49	817	1.46 (1.20-1.78)	<0.001	0.461
	≥60	24	696	1.03 (0.62-1.70)	0.909	0.461

Models adjust for: age, sex, smoking, hypertension, prior MI, prior stroke, multi−vessel disease, LM/LAD CTO, LDL−C, TG, NT-proBNP, TNT, creatinine, UA, CRP, LVEF, β‐Blocker, CCB, and ACEI/ARB.

HR, hazard ratio; CI, confidence interval; HGI, hemoglobin glycation index; CTO, chronic total occlusion; PCI, percutaneous coronary intervention.

### Sensitivity analyses

The robustness of our principal findings was reinforced by several sensitivity evaluations (**Supplementary Table 3**-**S7**). Across all sensitivity models, each 1-SD increment in HGI remained consistently associated with all-cause mortality (HRs 1.24–1.33; all P ≤ 0.026), cardiovascular mortality (HRs 1.37–1.50; all P ≤ 0.002), and CVEs (HRs 1.25–1.35; all P ≤ 0.027), thus confirming the stability of our main analyses.

## Discussion

Our analysis of 1, 513 patients from this single-center CTO-PCI registry demonstrates that higher HGI independently and linearly correlates with a greater burden of long-term adverse outcomes. After extensive adjustment for conventional risk factors, each 1-SD increase in HGI corresponded to a 34% higher risk of all-cause death, 52% higher cardiovascular death, and 36% higher CVEs. RCS analysis revealed a linear dose-response relationship between HGI and all three endpoints, with a threshold effect identified at HGI = -0.15 for CVEs. To our knowledge, this is the first report to systematically examine the prognostic role of HGI specifically in the CTO-PCI population, a group with unique pathophysiology involving chronic ischemia, collateralization, and heightened inflammation and thrombosis.

Our results align with and expand upon previous observations linking HGI to adverse cardiovascular prognosis across diverse CAD populations. Earlier reports have consistently demonstrated an independent association between HGI and major adverse events in patients with established coronary disease, often in a U-shaped fashion whereby both extremes of HGI confer excess risk (12–14, 21, 22). For instance, among 10, 598 post-PCI patients, those in the lowest HGI tertile faced significantly higher all-cause mortality (HR 1.68, 95% CI 1.18-2.40) and cardiovascular death (HR 1.60, 95% CI 1.06-2.42) relative to the middle tertile, whereas the highest tertile showed an elevated risk of major adverse cardiovascular events (HR 1.25, 95% CI 1.02-1.52) (13). Similarly, a separate cohort of 1, 780 CAD patients post-PCI reported that both low and high HGI subgroups had substantially increased all-cause mortality compared with the intermediate group (HR 4.98, 95% CI 1.87-13.30 and HR 2.92, 95% CI 1.08-7.92, respectively) (21). Taken together, these findings reinforce the prognostic significance of HGI as a marker of glycation heterogeneity that adds value beyond conventional glycaemic indices. However, our study differs from prior investigations in several important respects. First, we focused exclusively on patients with successful CTO revascularization, a cohort in whom procedural success has been achieved yet residual cardiovascular risk remains substantial. The distinct pathophysiology of CTO, characterized by chronic ischemia-induced hibernation, collateral circulation remodeling, and sustained inflammatory activation, may amplify the prognostic signal of glycation-related metabolic perturbation. Second, unlike the U-shaped relationships reported in general diabetic and CAD populations, we observed a linear association between HGI and all three clinical endpoints in our CTO PCI cohort. This divergence may reflect the distinct metabolic and inflammatory phenotype of patients undergoing CTO PCI, who typically exhibit more advanced atherosclerotic disease and greater physiologic perturbation than those undergoing non-CTO intervention. Alternatively, the smaller sample size and shorter follow-up duration in our cohort relative to prior studies may have contributed to this difference. Third, the magnitude of association observed in our study is notably larger than that reported in many prior studies, suggesting that HGI may serve as a particularly potent risk stratifier in this high-risk subgroup.

The strong and graded associations between HGI and adverse outcomes in our CTO-PCI cohort underscore the potential value of integrating glycation variability assessment into post-procedural risk stratification. Several mechanistic pathways may hypothetically underlie these observations; however, these mechanisms were not directly evaluated in our study and should be considered as speculative hypotheses warranting further investigation. First, HGI reflects the individual propensity for non-enzymatic protein glycation, a process that generates advanced glycation end-products (AGEs) (23, 24). AGEs accumulate in vascular tissues and promote atherosclerosis through multiple convergent mechanisms: cross-linking of extracellular matrix proteins, activation of the receptor for AGEs (RAGE) with downstream NF-κB–mediated inflammation, oxidative stress generation via NADPH oxidase activation, and modification of low-density lipoprotein to enhance foam cell formation (25–28). In the context of CTO, where chronic ischemia and collateral-dependent perfusion create a substrate of endothelial dysfunction and microvascular rarefaction, AGE-mediated vascular injury may be particularly deleterious (15, 29, 30). Second, elevated HGI indicates that observed HbA1c overestimates actual glycemic exposure, potentially leading to overly aggressive glucose-lowering therapy and consequent hypoglycemia risk. Hypoglycemia triggers sympathetic activation, catecholamine surge, and pro-arrhythmic effects, all of which may precipitate cardiovascular events in a population with already compromised coronary reserve (31, 32). Conversely, low HGI may reflect occult hyperglycemia masked by shortened erythrocyte survival or iron deficiency, permitting sustained tissue glycation and oxidative damage despite apparently satisfactory HbA1c levels. Third, HGI has been shown to correlate with systemic inflammatory markers—including CRP, erythrocyte sedimentation rate, complement C3, and white blood cell count—independent of HbA1c, suggesting that HGI captures a dimension of metabolic inflammation not reflected by conventional glycemic metrics (33). In the CTO setting, where chronic low-grade inflammation is a hallmark feature, this inflammatory pathway may be especially relevant.

The finding that HGI retained independent prognostic significance after adjustment for a comprehensive panel of cardiovascular risk factors, including LDL-C, NT-proBNP, TNT, creatinine, and LVEF, has important clinical implications. HbA1c, the cornerstone of glycemic monitoring, reflects average glucose exposure over approximately 120 days but fails to capture inter-individual variability in hemoglobin glycation at any given glucose level. This glycation variability is partly genetically determined—polymorphisms in genes encoding glycation-related enzymes such as fructosamine-3-kinase and amadoriase influence the rate of AGE formation—and partly modulated by factors including iron status, erythrocyte lifespan, and oxidative stress. By quantifying the discrepancy between observed and predicted HbA1c, HGI provides a personalized correction for this glycation bias and may identify patients in whom HbA1c either overestimates or underestimates true glycemic exposure and tissue glycation burden. From a practical standpoint, HGI can be calculated from routine laboratory measurements without additional cost or specialized equipment, suggesting that it may be a feasible adjunct for risk assessment in post-CTO-PCI surveillance, provided its prognostic utility is confirmed in prospective studies. The threshold effect analysis, which identified an cut-point at HGI= -0.15 for cardiovascular events, provides a potentially actionable benchmark for clinical decision-making. Patients with HGI above this threshold exhibited a 2.05-fold higher risk of cardiovascular events compared with those below it, suggesting that this cutoff may delineate a high-risk phenotype warranting intensified secondary prevention. Whether pharmacological or lifestyle interventions that specifically target glycation variability, such as strict glycemic control with agents that minimize glucose fluctuations, antioxidant therapy, or dietary AGE restriction, can modify the prognostic impact of HGI in CTO-PCI patients remains to be determined and should be explored in prospective interventional studies. However, the cut-point at HGI= -0.15 was derived from an exploratory analysis within a single cohort and should be considered hypothesis-generating rather than clinically definitive. External validation in independent, preferably multicenter, CTO-PCI populations is essential before this threshold can be applied to risk stratification or treatment decisions. We therefore urge caution in interpreting this specific value and recommend that future prospective studies specifically test the reproducibility and generalizability of this cut-off across diverse healthcare settings and ethnic populations.

This study has several strengths. First, to our knowledge, this is the first investigation to examine the association between HGI and long-term clinical outcomes in a large, well-characterized cohort of patients with successful CTO revascularization. Second, the single-center design with standardized protocols for angiographic assessment, laboratory analysis, and follow-up surveillance minimized interinstitutional variability and enhanced internal validity. Third, comprehensive adjustment for established cardiovascular risk factors strengthened causal inference. Fourth, the consistency of our findings across multiple sensitivity analyses—including exclusion of patients with follow-up shorter than 90 days, prior CABG, and severely reduced left ventricular ejection fraction—supports the reliability of the primary results.

Several limitations of this study warrant consideration. First, the retrospective, single-center design precludes causal inference and may constrain generalizability to diverse ethnic populations or healthcare settings. The HGI regression equation was derived from our specific cohort, following the established methodology in the HGI literature; this internal derivation, while standard practice, may limit generalizability to other populations and introduces the potential for optimism bias. External validation in independent, preferably multicenter, CTO-PCI cohorts is essential to confirm the generalizability of both the HGI equation and the prognostic associations observed in this study. Additionally, as with any observational study, the exclusion of patients due to procedural failure, incomplete data, or loss to follow-up carries the potential for selection bias. However, these exclusions were based on prespecified criteria, the proportion of excluded patients was modest (13.8%), and loss to follow-up was low (5.7%) and comparable to other contemporary CTO-PCI registries. We therefore believe that selection bias is unlikely to have substantially influenced our findings. Nevertheless, the potential for residual selection bias cannot be entirely excluded, and our results should be interpreted with this limitation in mind. Second, HGI was derived from a single baseline measurement of FPG and HbA1c, which did not allow us to capture longitudinal changes in glycemic status, the effects of glucose-lowering therapy intensification, or within-patient variability over time. Consequently, we cannot exclude the possibility that changes in HGI during follow-up might have influenced the observed associations. However, our sensitivity analysis excluding patients with follow-up <90 days yielded consistent results, reducing the likelihood that acute periprocedural glycemic fluctuations or reverse causality substantially biased our estimates. Future studies with serial HGI measurements are warranted to evaluate whether dynamic changes in HGI provide incremental prognostic information beyond baseline values. Third, DM status was defined using self-report, medication history, and laboratory criteria, but DM duration was not systematically recorded; consequently, we could not assess whether the observed associations differ by disease chronicity. Fourth, as in all observational research, residual confounding from unmeasured variables, including dietary habits, physical activity, treatment adherence, and genetic predisposition, cannot be entirely excluded. Fifth, the median follow-up of 810 days is relatively limited, which may have underestimated the incidence of late events and precluded analysis of very long-term outcomes. Sixth, the predominantly male composition of our cohort (91.6%), although consistent with the epidemiology of CTO-PCI, reduces the statistical power for sex-specific subgroup analyses. Seventh, the study period spanned the COVID-19 pandemic, which may have influenced patient management, follow-up intensity, and healthcare access. Although we attempted to assess this potential confounder, the relatively small number of outcome events precluded a robust stratified analysis by enrollment period (pre-pandemic vs. pandemic). The limited statistical power in period-specific subgroups would have produced unstable estimates and wide confidence intervals, making any meaningful comparison unreliable. We therefore acknowledge the potential for pandemic-related unmeasured confounding and encourage future large-scale studies to specifically evaluate the differential prognostic value of HGI across varying healthcare environments and periods. Eighth, although the number of events relative to covariates in Model 3 is modest, sensitivity analyses using a more parsimonious model yielded consistent results, with the HGI coefficient differing by less than 10% between the full and parsimonious models. This mitigates concerns about overfitting and model instability. Nonetheless, these findings should be confirmed in larger cohorts with greater event counts. Finally, the HGI computation presumes a linear relationship between FPG and HbA1c within the study sample; if this relationship is nonlinear or population-specific, measurement error could be introduced.

## Conclusion

In summary, among patients with successful CTO-PCI, higher HGI was independently and linearly associated with greater long-term mortality and cardiovascular morbidity. These findings suggest that HGI holds promise as a pragmatic, low-cost candidate marker for refining risk stratification in this high-risk population. However, given the observational design and single-center nature of our study, these results should be considered hypothesis-generating. Prospective multicenter investigations with external validation are urgently needed to confirm our observations and to determine whether HGI-guided risk modification strategies can improve patient outcomes.

## Data Availability

The raw data supporting the conclusions of this article will be made available by the authors, without undue reservation.
