# Supplementary material for: Hemoglobin glycation index and prognosis after successful chronic total occlusion percutaneous coronary intervention: a retrospective cohort study
Source: Front Endocrinol (Lausanne). 2026 Sep 10;17:1945216. doi: 10.3389/fendo.2026.1945216 (PMC13600852; doi:10.3389/fendo.2026.1945216)
Supplement: Supplementary file 1 [file Table1.docx]

**Table S1. Subgroup analysis of association of HGI and all-cause mortality in participants with successful CTO PCI.**

| **Subgroups** | | **All-cause mortality** | **N** | **HR(95%CI)** | **P value** | **P for interaction** |
| --- | --- | --- | --- | --- | --- | --- |
| Age | ≤60 | 26 | 814 | 1.61 (1.12-2.31) | 0.010 | 0.415 |
|  | >60 | 57 | 699 | 1.31 (1.05-1.64) | 0.018 | 0.415 |
| Diabetes | Yes | 44 | 602 | 1.28 (1.03-1.57) | 0.023 | 0.228 |
|  | No | 39 | 911 | 1.87 (1.09-3.21) | 0.022 | 0.228 |
| Hypertension | Yes | 62 | 902 | 1.25 (1.02-1.53) | 0.029 | 0.113 |
|  | No | 21 | 611 | 1.87 (1.28-2.75) | 0.001 | 0.113 |
| Prior MI | Yes | 21 | 237 | 1.50 (0.92-2.44) | 0.108 | 0.398 |
|  | No | 62 | 1,276 | 1.32 (1.09-1.59) | 0.005 | 0.398 |
| Current Smoking | Yes | 28 | 588 | 1.32 (0.91-1.92) | 0.143 | 0.808 |
|  | No | 55 | 925 | 1.36 (1.12-1.65) | 0.002 | 0.808 |
| LDL-C | ≥2.6 | 33 | 590 | 1.22 (0.95-1.56) | 0.120 | 0.561 |
|  | <2.6 | 50 | 923 | 1.48 (1.12-1.96) | 0.006 | 0.561 |
| LVEF | <60 | 63 | 817 | 1.34 (1.12-1.61) | 0.002 | 0.856 |
|  | ≥60 | 20 | 696 | 1.16 (0.67-2.01) | 0.585 | 0.856 |

Models adjust for: age, sex, smoking, hypertension, prior MI, prior stroke, multi‑vessel disease, LM/LAD CTO, LDL‑C, TG, NT-proBNP, TNT, creatinine, UA, CRP, LVEF, β‐Blocker, CCB, and ACEI/ARB.

HR, hazard ratio; CI, confidence interval; HGI, hemoglobin glycation index; CTO, chronic total occlusion; PCI, percutaneous coronary intervention.

**Table S2. Subgroup analysis of association of HGI and cardiovascular mortality in participants with successful CTO PCI.**

| **Subgroups** | | **Cardiovascular Mortality** | **N** | **HR (95%CI)** | **P value** | **P for interaction** |
| --- | --- | --- | --- | --- | --- | --- |
| Age | ≤60 | 17 | 814 | 1.94 (1.28-2.94) | 0.002 | 0.200 |
|  | >60 | 36 | 699 | 1.54 (1.17-2.01) | 0.002 | 0.200 |
| Diabetes | Yes | 30 | 602 | 1.48 (1.17-1.88) | <0.001 | 0.424 |
|  | No | 23 | 911 | 1.91 (0.86-4.26) | 0.113 | 0.424 |
| Hypertension | Yes | 40 | 902 | 1.42 (1.11-1.82) | 0.005 | 0.046 |
|  | No | 13 | 611 | 2.10 (1.50-2.96) | <0.001 | 0.046 |
| Prior MI | Yes | 16 | 237 | 1.68 (1.00-2.83) | 0.049 | 0.784 |
|  | No | 37 | 1,276 | 1.53 (1.22-1.92) | <0.001 | 0.784 |
| Current Smoking | Yes | 21 | 588 | 1.40 (0.90-2.16) | 0.133 | 0.950 |
|  | No | 32 | 925 | 1.68 (1.32-2.13) | <0.001 | 0.950 |
| LDL-C | ≥2.6 | 23 | 590 | 1.30 (0.99-1.72) | 0.061 | 0.392 |
|  | <2.6 | 30 | 923 | 1.79 (1.27-2.53) | <0.001 | 0.392 |
| LVEF | <60 | 43 | 817 | 1.54 (1.25-1.89) | <0.001 | 0.885 |
|  | ≥60 | 10 | 696 | 1.25 (0.50-3.13) | 0.639 | 0.885 |

Models adjust for: age, sex, smoking, hypertension, prior MI, prior stroke, multi‑vessel disease, LM/LAD CTO, LDL‑C, TG, NT-proBNP, TNT, creatinine, UA, CRP, LVEF, β‐Blocker, CCB, and ACEI/ARB.

HR, hazard ratio; CI, confidence interval; HGI, hemoglobin glycation index; CTO, chronic total occlusion; PCI, percutaneous coronary intervention.

Table S3. Associations between HGI and clinical outcomes in participants with successful CTO PCI after excluded participants with LVEF＜30% (N = 1434)

|  |  | **All-cause mortality** | |  | **Cardiovascular mortality** | |  | **Cardiovascular events** | |
| --- | --- | --- | --- | --- | --- | --- | --- | --- | --- |
|  |  | **HR (95%CI)** | ***P*-value** |  | **HR (95%CI)** | ***P*-value** |  | **HR (95%CI)** | ***P*-value** |
| Per 1-SD increase in HGI |  | 1.26 (1.05-1.51) | 0.013 |  | 1.42 (1.13-1.77) | 0.002 |  | 1.25 (1.03-1.52) | 0.022 |
| T1 |  | Reference |  |  | Reference |  |  | Reference |  |
| T2 |  | 1.35 (0.65-2.82) | 0.422 |  | 1.73 (0.64-4.65) | 0.277 |  | 1.40 (0.65-3.01) | 0.393 |
| T3 |  | 2.36 (1.25-4.45) | 0.008 |  | 4.07 (1.72-9.63) | 0.001 |  | 2.44 (1.26-4.74) | 0.008 |

Models adjust for: age, sex, smoking, hypertension, prior MI, prior stroke, multi‑vessel disease, LM/LAD CTO, LDL‑C, TG, NT-proBNP, TNT, creatinine, UA, CRP, LVEF, β‐Blocker, CCB, and ACEI/ARB.

HR, hazard ratio; CI, confidence interval; HGI, hemoglobin glycation index; CTO, chronic total occlusion; PCI, percutaneous coronary intervention.

Table S4. Associations between HGI and clinical outcomes in participants with successful CTO PCI after excluded participants with prior CABG (N = 1477)

|  |  | **All-cause mortality** | |  | **Cardiovascular mortality** | |  | **Cardiovascular events** | |
| --- | --- | --- | --- | --- | --- | --- | --- | --- | --- |
|  |  | **HR (95%CI)** | ***P*-value** |  | **HR (95%CI)** | ***P*-value** |  | **HR (95%CI)** | ***P*-value** |
| Per 1-SD increase in HGI |  | 1.33 (1.13-1.57) | <0.001 |  | 1.50 (1.24-1.81) | <0.001 |  | 1.35 (1.14-1.60) | <0.001 |
| T1 |  | Reference |  |  | Reference |  |  | Reference |  |
| T2 |  | 1.00 (0.51-2.00) | 0.989 |  | 1.45 (0.57-3.68) | 0.433 |  | 1.25 (0.60-2.63) | 0.548 |
| T3 |  | 2.28 (1.29-4.01) | 0.004 |  | 4.11 (1.87-9.03) | <0.001 |  | 2.71 (1.45-5.07) | 0.002 |

Models adjust for: age, sex, smoking, hypertension, prior MI, prior stroke, multi‑vessel disease, LM/LAD CTO, LDL‑C, TG, NT-proBNP, TNT, creatinine, UA, CRP, LVEF, β‐Blocker, CCB, and ACEI/ARB.

HR, hazard ratio; CI, confidence interval; HGI, hemoglobin glycation index; CTO, chronic total occlusion; PCI, percutaneous coronary intervention.

Table S5. Associations between HGI and clinical outcomes in participants with successful CTO PCI after excluded participants with follow-up≤90 day (N = 1477)

|  |  | **All-cause mortality** | |  | **Cardiovascular mortality** | |  | **Cardiovascular events** | |
| --- | --- | --- | --- | --- | --- | --- | --- | --- | --- |
|  |  | **HR (95%CI)** | ***P*-value** |  | **HR (95%CI)** | ***P*-value** |  | **HR (95%CI)** | ***P*-value** |
| Per 1-SD increase in HGI |  | 1.26 (1.03-1.54) | 0.026 |  | 1.45 (1.14-1.84) | 0.002 |  | 1.26 (1.03-1.55) | 0.027 |
| T1 |  | Reference |  |  | Reference |  |  | Reference |  |
| T2 |  | 0.93 (0.43-2.04) | 0.862 |  | 1.62 (0.51-5.21) | 0.416 |  | 1.28 (0.55-2.98) | 0.568 |
| T3 |  | 2.17 (1.15-4.08) | 0.016 |  | 4.64 (1.74-12.37) | 0.002 |  | 2.63 (1.29-5.35) | 0.008 |

Models adjust for: age, sex, smoking, hypertension, prior MI, prior stroke, multi‑vessel disease, LM/LAD CTO, LDL‑C, TG, NT-proBNP, TNT, creatinine, UA, CRP, LVEF, β‐Blocker, CCB, and ACEI/ARB.

HR, hazard ratio; CI, confidence interval; HGI, hemoglobin glycation index; CTO, chronic total occlusion; PCI, percutaneous coronary intervention.

Table S6. Associations between HGI and clinical outcomes after successful CTO‑PCI (adjusted for parsimonious model covariates) (N = 1513).

|  |  | **All-cause mortality** | |  | **Cardiovascular mortality** | |  | **Cardiovascular events** | |
| --- | --- | --- | --- | --- | --- | --- | --- | --- | --- |
|  |  | **HR (95%CI)** | ***P*-value** |  | **HR (95%CI)** | ***P*-value** |  | **HR (95%CI)** | ***P*-value** |
| Per 1-SD increase in HGI |  | 1.24 (1.05-1.46) | 0.013 |  | 1.37 (1.14-1.66) | 0.001 |  | 1.31 (1.10-1.57) | 0.003 |
| T1 |  | Reference |  |  | Reference |  |  | Reference |  |
| T2 |  | 0.97 (0.50-1.90) | 0.933 |  | 1.28 (0.52-3.15) | 0.586 |  | 1.16 (0.56-2.37) | 0.692 |
| T3 |  | 2.31 (1.28-4.18) | 0.006 |  | 3.74 (1.69-8.23) | <0.001 |  | 2.68 (1.41-5.09) | 0.003 |

Models adjust for: age, hypertension, diabetes, prior stroke, NT-proBNP, TNT, creatinine, UA, CRP, and LVEF.

HR, hazard ratio; CI, confidence interval; HGI, hemoglobin glycation index; CTO, chronic total occlusion; PCI, percutaneous coronary intervention.

Table S7. Associations between HGI and clinical outcomes in participants with successful CTO PCI additionally adjusted for diabetes (N = 1513).

|  |  | **All-cause mortality** | |  | **Cardiovascular mortality** | |  | **Cardiovascular events** | |
| --- | --- | --- | --- | --- | --- | --- | --- | --- | --- |
|  |  | **HR (95%CI)** | ***P*-value** |  | **HR (95%CI)** | ***P*-value** |  | **HR (95%CI)** | ***P*-value** |
| Per 1-SD increase in HGI |  | 1.33 (1.11-1.59) | 0.002 |  | 1.50 (1.22-1.84) | <0.001 |  | 1.35 (1.12-1.62) | 0.001 |
| T1 |  | Reference |  |  | Reference |  |  | Reference |  |
| T2 |  | 1.07 (0.54-2.12) | 0.845 |  | 1.43 (0.57-3.58) | 0.445 |  | 1.22 (0.59-2.54) | 0.588 |
| T3 |  | 2.50 (1.37-4.55) | 0.003 |  | 4.38 (1.96-9.80) | <0.001 |  | 2.81 (1.46-5.39) | 0.002 |

Models adjust for: age, sex, smoking, hypertension, prior MI, prior stroke, diabetes, multi‑vessel disease, LM/LAD CTO, LDL‑C, TG, NT-proBNP, TNT, creatinine, UA, CRP, LVEF, β‐Blocker, CCB, and ACEI/ARB.

HR, hazard ratio; CI, confidence interval; HGI, hemoglobin glycation index; CTO, chronic total occlusion; PCI, percutaneous coronary intervention.
